# Delayed neutrophil apoptosis may enhance NET formation in ARDS

**DOI:** 10.1186/s12931-022-02065-y

**Published:** 2022-06-13

**Authors:** Chao Song, Haitao Li, Zhi Mao, Ling Peng, Ben Liu, Fengyu Lin, Yi Li, Minhui Dai, Yanhui Cui, Yuhao Zhao, Duoduo Han, Lingli Chen, Xun Huang, Pinhua Pan

**Affiliations:** 1 Infection Control Center, Xiangya Hospital, Central South University, 87 Xiangya Road, Kaifu District, Changsha, 410008 Hunan China; 2 Department of Respiratory Medicine, National Key Clinical Specialty, Branch of National Clinical Research Center for Respiratory Disease, Xiangya Hospital, Central South University, Changsha, 410008 Hunan China; 3 Cancer Hospital of Hunan Province, Changsha, 410006 Hunan China; 4 Shenzhen Third People’s Hospital of Guangdong Province, Shenzhen, 518114 Guangdong China; 5 National Clinical Research Center for Geriatric Disorders, Xiangya Hospital, Central South University, Changsha, 410008 Hunan China; 6 Present Address: Center of Respiratory Medicine, Xiangya Hospital, Central South University, Changsha, 410008 Hunan China

**Keywords:** Neutrophil apoptosis, Neutrophil extracellular traps, CDK inhibitor, Acute respiratory distress syndrome

## Abstract

**Background:**

Acute respiratory distress syndrome (ARDS) is a neutrophil-associated disease. Delayed neutrophil apoptosis and increased levels of neutrophil extracellular traps (NETs) have been described in ARDS. We aimed to investigate the relationship between these phenomena and their potential as inflammation drivers. We hypothesized that delayed neutrophil apoptosis might enhance NET formation in ARDS.

**Method:**

Our research was carried out in three aspects: clinical research, animal experiments, and in vitro experiments. First, we compared the difference between neutrophil apoptosis and NET levels in healthy controls and patients with ARDS and analyzed the correlation between neutrophil apoptosis and NET levels in ARDS. Then, we conducted animal experiments to verify the effect of neutrophil apoptosis on NET formation in Lipopolysaccharide-induced acute lung injury (LPS-ALI) mice. Furthermore, this study explored the relationship between neutrophil apoptosis and NETs at the cellular level. Apoptosis was assessed using morphological analysis, flow cytometry, and western blotting. NET formation was determined using immunofluorescence, PicoGreen assay, SYTOX Green staining, and western blotting.

**Results:**

ARDS neutrophils lived longer because of delayed apoptosis, and the cyclin-dependent kinase inhibitor, AT7519, reversed this phenomenon both in ARDS neutrophils and neutrophils in bronchoalveolar lavage fluid (BALF) of LPS-ALI mice. Neutrophils in a medium containing pro-survival factors (LPS or GM-CSF) form more NETs, which can also be reversed by AT7519. Tissue damage can be reduced by promoting neutrophil apoptosis.

**Conclusions:**

Neutrophils with extended lifespan in ARDS usually enhance NET formation, which aggravates inflammation. Enhancing neutrophil apoptosis in ARDS can reduce the formation of NETs, inhibit inflammation, and consequently alleviate ARDS.

**Supplementary Information:**

The online version contains supplementary material available at 10.1186/s12931-022-02065-y.

## Background

Acute respiratory distress syndrome (ARDS) is a clinical condition characterized by extensive inflammatory reactions in the lungs, usually secondary to pneumonia, sepsis, and trauma. The mortality of patients with ARDS is between 20 and 50% [1], with no effective pharmacological therapies in routine clinical practice that target the dysregulated and overwhelming inflammatory response, which is a characteristic of ARDS [2].

Neutrophils play a central role in the initiation, spread, and resolution of complex inflammatory environments by migrating into the lungs and performing various pro-inflammatory functions in ARDS. These include release of bactericidal proteins, cytokines, and reactive oxygen species, and production of neutrophil extracellular traps (NETs). Although these functions are beneficial in clearing bacterial inflammation, the prolongation of neutrophil life at the inflammation sites is deleterious [3, 4].

Neutrophils are short-lived cells that undergo caspase-dependent apoptosis within a few hours. The efferocytosis of apoptotic neutrophils by macrophages promotes anti-inflammatory signaling, prevents neutrophil lysis, and dampens immune responses, as a result, promoting the elimination of inflammation and restore tissue homeostasis [2, 5]. The survival of neutrophils is controlled by pro-apoptotic and anti-apoptotic factors in the cell’s internal and external environment. Granulocyte-macrophage colony-stimulating factor (GM-CSF) [6], tumor necrosis factor (TNF), hypoxia, and bacterial endotoxin can all prolong the life of neutrophils by increasing the protein levels of Bcl-2 family member, Mcl-1. Therefore, spontaneous apoptosis is delayed in various diseases (such as ARDS, cystic fibrosis, and sepsis), causing persistent inflammatory responses and tissue damage [7, 8]. Our previous study has shown that patients with ARDS usually produce excessive NETs [9]. While exerting their bactericidal and anti-infective effects, they also promote macrophage polarization to M1 phenotype, aggravating the inflammatory response and tissue damage in patients with ARDS [10].

Some studies have pointed out that delayed neutrophil apoptosis causes increased production of NETs, which aggravates the inflammatory response. The acceleration of apoptosis can improve oxygenation and reduce inflammation, and accelerate inflammation resolution in preclinical models [6, 8, 11].

Even though human neutrophils do not proliferate or divide, the proteins they express are often involved in cell cycle progression and mitosis [12, 13]. Human neutrophils express CDK-2, -7, and -9 (plus several others at low expression levels), and inhibition of these kinases can lead to accelerated apoptosis [14–16]. The CDK inhibitor AT7519 can induce apoptosis via the activation of GSK-3β, and this protein can reduce Mcl-1 (myeloid cell leukemia-1) levels after activation [17, 18]. A study has demonstrated that the inhibition or knockdown of CDK4/6 inhibits the generation of NETs [19]. Thus, CDK expression may serve as a target for the management of inflammatory diseases.

Here, we hypothesized that delayed neutrophil apoptosis might enhance NET formation in ARDS and that the CDK inhibitor, AT7519, would promote neutrophil apoptosis to reduce the NET formation, thus limiting inflammation and tissue damage.

## Methods

### Human research subjects

In accordance with the Berlin Definition of ARDS [20], patients with ARDS caused by gram-negative bacterial pneumonia were enrolled from the respiratory intensive care unit (RICU) of Xiangya Hospital. We collected samples from patients within 48 h of ARDS diagnosis. The exclusion criteria were: < 18-year-old, pregnant, and septic, aspiration, and other non-bacterial forms of ARDS. Healthy volunteers were used as the control group. The Medical Ethics Committee of Xiangya Hospital approved the research (IRB{S}NO.2017121025). Since this research builds upon our published research results, they share the same ethical number. Written informed consent was obtained from the relatives of patients and healthy volunteers.

According Berlin Definition [20], ARDS were categorized as having “mild”, “moderate” or “severe” ARDS based on the ratio of the PaO_2_/FiO_2_, namely oxygenation index (mild: 200 < PaO_2_/FiO_2_ ≤ 300 mmHg; moderate: 100 < PaO_2_/FiO_2_ ≤ 200 mmHg; severe: PaO_2_/FiO_2_ ≤ 100 mmHg)**.** We obtained the peripheral blood of 26 ARDS patients and 21 healthy controls for the detection of NET levels in plasma. Among them, the number of ARDS patients used for neutrophil apoptosis detection was 22, the healthy control group was 13, and the age of 22 ARDS patients was 65.46 ± 16.02, the male to female ratio was 13:9. 11 patients were mild ARDS, and there were 9 patients with moderate degree and 2 with severe degree. The age of the 13 control groups was 58.15 ± 16.50, male to female was 6:7, and there was no statistical difference in age and gender for the 22 patients and 13 controls. Details were listed in Additional file 1: Table S1. Besides, the specific neutrophil apoptosis rate, oxygenation index and the corresponding APACHE II score were showed in Additional file 2: Table S2.

### Collection of samples from patients with ARDS

Within 48 h of ARDS diagnosis, 4 ml of venous blood was collected and centrifuged at 3000 rpm for 10 min at 4 ℃, and the plasma was stored at − 80 °C. Neutrophils were isolated using a EasySep™ Direct Human Neutrophil Isolation Kit (STEMCELL Technologies, Vancouver, Canada) following the instructions of the manufacturer. Isolated cells were washed twice in cation-free Dulbecco’s phosphate-buffered saline (DPBS) and then resuspended in appropriate culture media for subsequent experiments.

### Human cell apoptosis measurement

The isolated neutrophils (1 × 10^6^/ml) were seeded in a 6-well plate and cultured with Iscove’s modified Dulbecco’s medium containing 10% fetal bovine serum (FBS) in the presence or absence of AT7519, GM-CSF, and lipopolysaccharide (LPS) (*Escherichia coli* 0111: B4; Sigma-Aldrich, St. Louis, MO, USA) for 24 h at 37 °C and 5% CO_2_. At 0 h and 24 h later, neutrophils were collected and washed once with DPBS. The neutrophils pellet was marked with CD45^+^CD66b^+^CD16^+^. Cell apoptosis was detected using the Annexin V-FITC/PI Apoptosis Detection kit (KeyGEN, China) following the manufacturer’s protocol. The neutrophils were resuspended with an apoptotic buffer and then labeled with Annexin V-FITC and PI for 15 min at 22–25 °C in the dark. Finally, the cells were analyzed using flow cytometry within 1 h (Becton Dickinson) (Ex 488 nm, Em 530 nm). Neutrophil smears were made, and the cells were stained with Diff-Quik (Solarbio, China) to assess the morphological changes of apoptosis.

### Western blotting

Western blotting was carried out as previously described [17], with the following antibodies: Mcl-1 (1:1000; Genetex, USA), cleaved-caspase 3 (1:1000; Cell Signaling Technology, USA), p-GSK-3β (Ser9) (1:1000; Cell Signaling Technology, USA), citrullinated histone H3 (Cit-H3, 1:1000; Abcam, USA), and GAPDH (1:5000; Signalway Antibody, Maryland, USA). ImageJ was used to calculate the grayscale values.

### Microscopic detection of NETs

Neutrophils were seeded (5 × 10^4^/well) into 24-well plates in DMEM with 10% FBS, with or without 1 µM AT7519 or 2.5 ng/ml GM-CSF for 8 h, followed by stimulation with 40 nM para-methoxyamphetamine (PMA) for 4 h at 37 °C and 5% CO_2_. After incubation, 0.15 µM SYTOX Green (Invitrogen, Thermo Scientific, UK) was added before capturing fluorescent images using the EVOS FL cell imaging system. SYTOX Green nucleic acid stain is an excellent green-fluorescent nuclear and chromosome counterstain that is impermeant to live cells but penetrates the compromised membranes characteristic of dead cells, making it a useful indicator of dead cells within a population. When the cell produces NETs, the nuclear membrane ruptures and DNA is released, so it can be stained by SYTOX Green. SYTOX Green-positive cells are neutrophils that produce NETs. We used ImageJ to quantify the cells in each field. The number of cells producing NETs in each group was the average number of cells obtained from five fields. All samples were plated in duplicate, and five zones were counted per well.

### Quantification of plasma cell-free DNA bound to myeloperoxidase levels

The concentration of cell-free DNA (cf-DNA) bound to myeloperoxidase (MPO) (cf-DNA/MPO), a composition of NET, was measured in the plasma. Briefly, the MPO protein was captured by antibodies bound to a 96-well flat-bottom plate, and the amount of DNA bound to the MPO protein was quantified using the Quant-iT™ PicoGreen Kit (Invitrogen).

### Identification of NETs

Neutrophils from healthy volunteers and patients with ARDS were seeded on 24-well coverslips and allowed to adhere for 1 h, then gently aspirated the supernatant and washed it with PBS, followed by stimulation with 10 nM PMA for 0.5 h at 37 °C and 5% CO_2_ to detcet whether the neutrophils of ARDS patients will be more sensitive to PMA stimulation. Then fixed with 4% paraformaldehyde for 30 min, permeabilized with 0.1% Triton X-100 for 10 min, and blocked with 5% goat serum for 1 h. The coverslips were incubated with Cit-H3 (1:100; Abcam) and MPO (1:100; R&D Systems) primary antibodies, followed by detection with FITC-conjugated IgG (1:400; Servicebio, Wuhan, Hubei, China) and Cy3-conjugated IgG (1:400; Servicebio) secondary antibodies, respectively, at 25 °C for 1 h. DAPI was used to visualize DNA, and the slides were observed using an Olympus FluoView 500 confocal microscope.

### In vivo experiments

#### Mice

C57BL/6 mice, aged 8–12 weeks, were purchased from the Experimental Animal Center of Central South University (Changsha, China). Mice were housed in cages under specific pathogen-free conditions. All animal experiments were approved by the Animal Care and Use Committee of Central South University (No.2017sydw00284).

#### Drug administration and specimens collection

In keeping with our specimens of ARDS caused by gram-negative bacteria pneumonia, we used LPS intratracheal administration to induce acute lung injury (ALI) in mice to simulate ARDS disease. ALI/ARDS mouse models were established as previously described [10]. Mice were divided into control group, LPS group and neutrophil apoptosis-promoting group (LPS + AT7519). For the group that promoted neutrophil apoptosis, LPS (3 mg/kg) in 60 μl of phosphate-buffered saline (PBS) was injected into the trachea on the 1st day of modeling, and then AT7519 (30 mg/kg) in 200 μl of PBS was injected intraperitoneally 24 h later. The control group received an intratracheal injection of 60 μl of PBS.

Mice were sacrificed 48 h later, the lungs were lavaged 3 times with 0.5 ml of PBS-EDTA (ethylenediamine tetraacetic acid) (0.5 mM) for the detection of neutrophils apoptosis, cell counts and differences in total protein. The left lungs were quickly isolated on ice and then stored in – 80 °C for western blotting. Right lungs were fixed with 4% paraformaldehyde for hematoxylin–eosin (HE) staining.

#### HE staining

For histopathological analysis, the fixed right lung was embedded in paraffin. Four micron sections were placed on glass slides and stained with HE. Briefly, the lung tissue injury index included pulmonary edema, hemorrhage, neutrophil infiltration and alveolar septal thickening [21]. Each item was divided into four grades ranging from 0 to 3 (0 = normal; 1 = mild; 2 = moderate; and 3 = severe), and a total ALI score was then calculated [10].

#### Quantification of protein and cf-DNA/MPO levels in mice bronchoalveolar lavage fluid

The total protein concentration in the BALF was detected by bicinchoninic acid protein assay (KeyGen Biotech, China), and the cf-DNA/MPO concentration measurement method was the same as above.

#### Mice neutrophil apoptosis measurement

Since more than 95% of the alveolar cells of normal mice are macrophages, and the proportion of neutrophils is very low, in animal experiments, we only compared the neutrophil apoptosis levels in BALF of the LPS group with the LPS + AT7519 group. Due to the small total volume of peripheral blood in mice, the number of neutrophils is not large, which is not an ideal sample source for detecting neutrophil apoptosis. BALF was centrifuged at 1000 rpm for 5 min at 4 °C, then washed twice with DPBS, then neutrophils were marked with CD45+LY6G+, Annexin-V and PI were labeled as described above, and the apoptosis of neutrophils in BALF was detected by flow cytometry. Each flow cytometric analysis was run on at least 100,000 cells, and the data were analyzed using FlowJo X.

### Statistical analyses

All data sets are expressed as mean ± Standard Error (SE), and p values of < 0.05 were considered as statistically significant differences. Differences among three or more groups were assessed using one-way ANOVA with Tukey’s post hoc test, and differences between two groups were assessed using the two-tailed Student’s t-test. The tests were performed using GraphPad Prime 9.0 software (GraphPad, San Diego, CA, USA).

## Results

### Apoptosis of neutrophils decreases in patients with ARDS

**Fig. 1:**
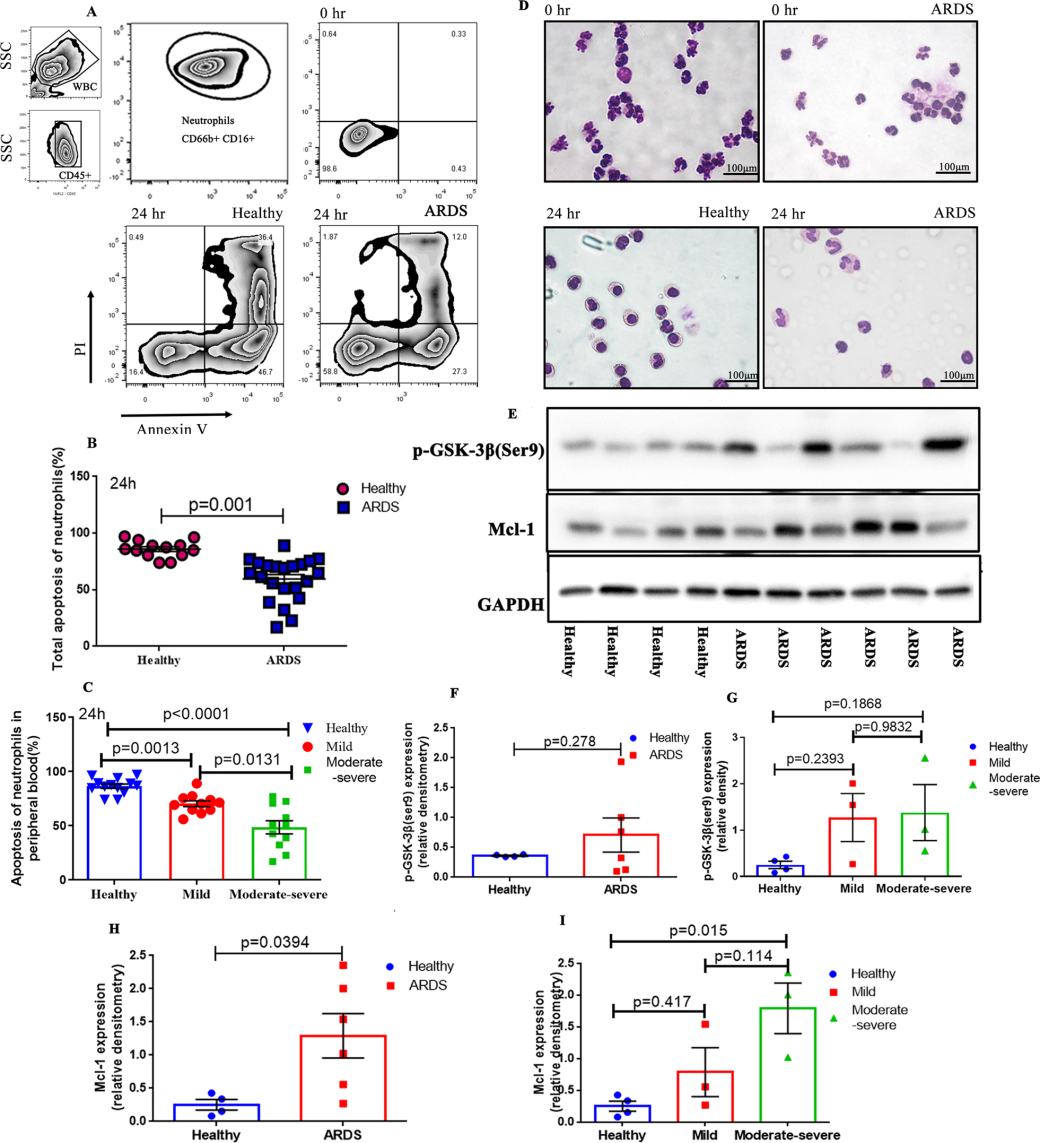
Apoptosis of neutrophils decreases in patients with acute respiratory distress syndrome (ARDS). **A**–**C** Flow cytometry; peripheral blood neutrophils from patients with ARDS (n = 22) displayed less apoptosis compared to those from healthy controls (n = 13) at 24 h of culture, and the rate of neutrophil apoptosis was further reduced with increasing disease severity. **D** Diff-Quik staining (400× magnification). **E–I** Western blotting to compare the protein levels of p-GSK-3β (Ser9) and Mcl-1 in patients with ARDS (n = 6) and healthy controls (n = 4). The immunoblot data currently presented as separate groups were run on the same gel (with non-essential lanes removed). p values < 0.05 indicated statistical significance

The short-lived prosurvival Bcl-2 family protein, Mcl-1, is instrumental in controlling apoptosis and consequently neutrophil lifespan in response to rapidly changing environmental cues during inflammation. Venous blood from healthy volunteers and patients with ARDS (within 48 h) diagnosed in the RICU of Xiangya Hospital was collected. In this part, we found that unstimulated peripheral blood neutrophils from patients with ARDS displayed less apoptosis than healthy controls at 24 h of culture (Fig. 1A and B; p = 0.001), moreover, patients with moderate-severe ARDS had the lowest apoptosis rate (Fig. 1C, healthy vs mild: p = 0.0013, mild vs moderate–severe: p = 0.0131). Diff-Quik staining (Fig. 1D) showed that cell morphology results were consistent with flow cytometry results. From the western blotting results (Fig. 1E–I), we know that the protein levels of Mcl-1 in patients with ARDS espicially the moderate-severe ARDS were higher than those in the control group, and the difference was statistically significant (Fig. 1H, Mcl-1; ARDS vs Healthy: p = 0.0394, moderate–severe vs healthy: p = 0.015). Although the quantification of western blotting showed there was no significance between healthy controls and mild ARDS of Mcl-1 (Fig. 1I, p = 0.417), its expression level directly increased with increasing ARDS severity. p-GSK-3β (Ser9) level was not significantly different between healthy people and patients with ARDS and did not appear to be affected by the severity of ARDS, but we found that the expression of p-GSK-3β (Ser9) in 3 out of 6 patients was significantly higher than that in healthy people. Because p-GSK-3β (Ser9) is related to inhibition of apoptosis [22], we will further elucidate its relationship with delayed neutrophil apoptosis in patients with ARDS using in vitro experiments. In in vitro experiments, we will use apoptosis inhibitors or drugs that promote apoptosis to interfere with neutrophils, and at the same time detect the level of p-GSK-3β. If the level of p-GSK-3β also changes, it will show that it is involved in the regulation of neutrophil apoptosis.

### Apoptosis of neutrophil negatively correlates with NET levels in the plasma of patients with ARDS

**Fig. 2:**
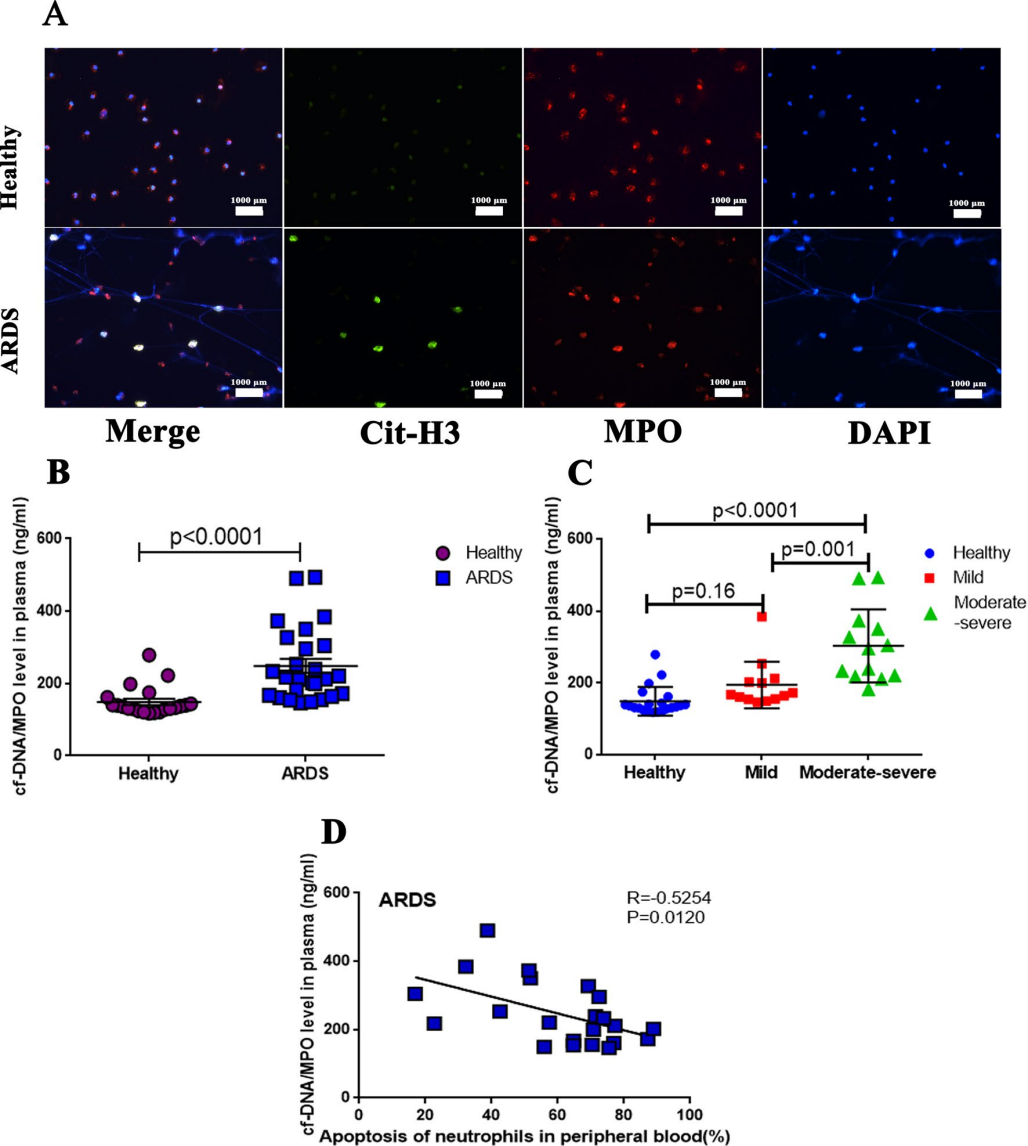
Apoptosis of neutrophil negatively correlates with neutrophil extracellular trap (NET) levels in the plasma of patients with acute respiratory distress syndrome (ARDS). **A** Immunofluorescence; **B**, **C** cf-DNA/MPO level was measured in the plasma of healthy volunteers (n = 21) and patients with ARDS (n = 26). **D** Pearson’s correlation analysis. Pearson’s correlation analysis showed that neutrophil apoptosis rate in patients with ARDS was negatively correlated with cf-DNA/MPO levels, n = 22. p values < 0.05 indicated statistical significance

cf-DNA and MPO are the main components of NETs, and the detection of the complex of the two can indirectly reflect the level of NETs [23]. We seeded 1 × 10^5^/ml neutrophils on 24-well coverslips. After the neutrophils adhered to coverslips for 1 h, they were stimulated with 10 nM PMA to produce NETs. The immunofluorescence results showed that neutrophils from ARDS patients could produce NETs when stimulated for 30 min, whereas the cells from the healthy people have no apparent NET formation at 30 min (Fig. 2A). Then, we used PicoGreen to test the differences in plasma cf-DNA/MPO levels between healthy volunteers and patients with ARDS. The cf-DNA/MPO levels in the plasma of patients with ARDS were significantly higher than those in the healthy group (Fig. 2B; p < 0.0001), and with the increase of ARDS severity, the cf-DNA/MPO levels also increased (Fig. 2C; moderate–severe vs mild: p = 0.001). These results indicated that neutrophils from patients with ARDS might be more likely to produce NETs than healthy persons, and the NETs levels are related to the severity of ARDS.

Pearson’s correlation analysis showed that neutrophil apoptosis rate in patients with ARDS was negatively correlated with cf-DNA/MPO levels (Fig. 2D; R = − 0.5254, p = 0.0120), which means that ARDS patients with high apoptosis rates had lower levels of NETs in plasma.

### Delayed neutrophil apoptosis and increased NET levels are positively correlated with ARDS

**Fig. 3:**
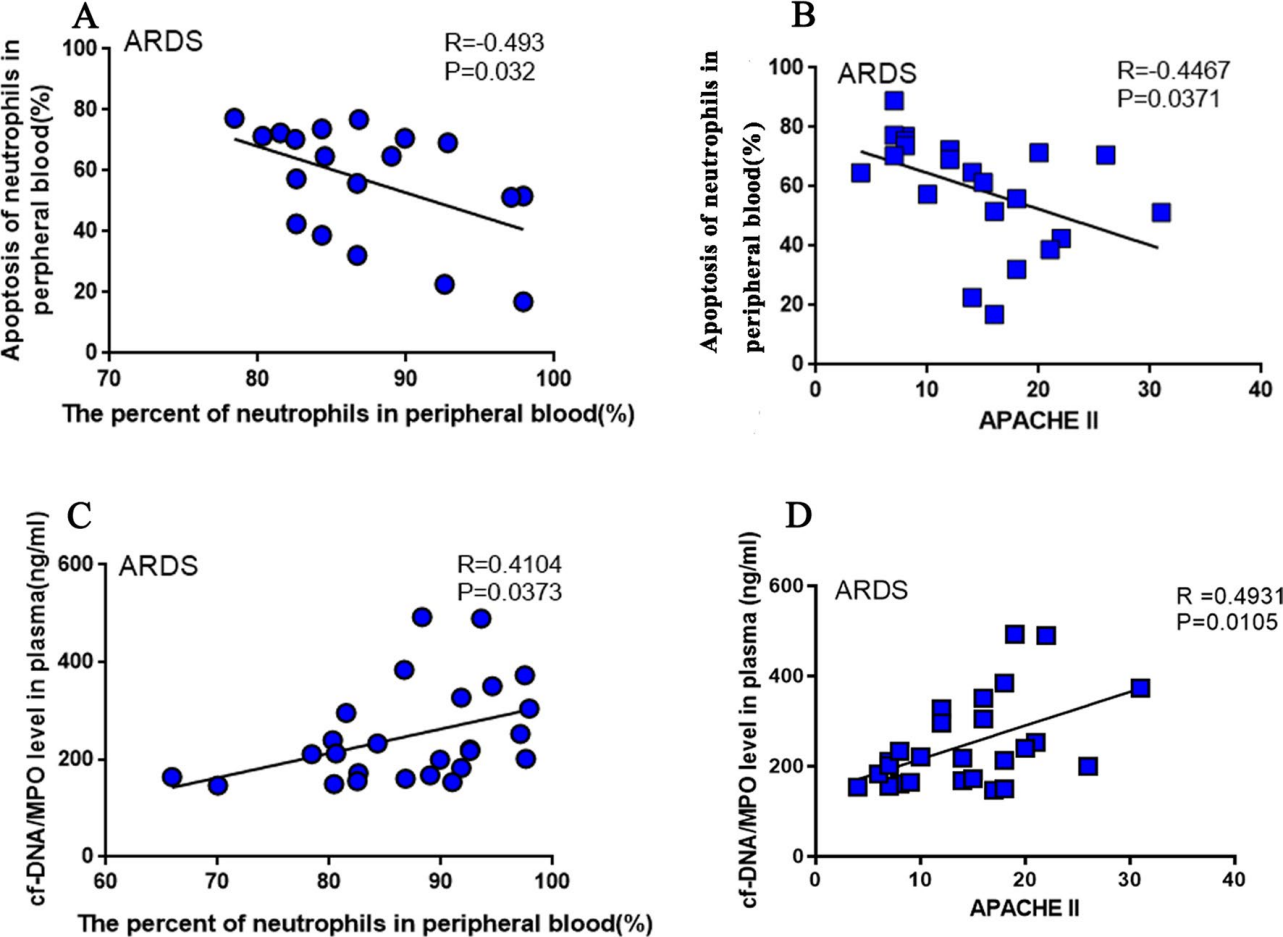
Delayed neutrophil apoptosis and increased neutrophil extracellular trap (NET) levels are positively correlated with acute respiratory distress syndrome (ARDS). **A**–**D** Pearson’s correlation analysis. **A** n = 19, remove 1 outlier; **B** n = 22; **C** and **D** n = 26. p values < 0.05 indicated statistical significance

Also, we recorded the proportion of neutrophils within 48 h when patients were diagnosed with ARDS. We found that the neutrophil apoptosis rate decreased as the proportion of neutrophils in peripheral blood increased (the date of routine blood sampling must be the same as the date of blood sample collection for apoptosis detection, and 1 outlier was removed, 19 ARDS patients were finally included to study the correlation between the proportion of neutrophils and apoptosis, Fig. 3A; R = − 0.493, p = 0.032). By analyzing the above data and the clinical data collected, we found that the degree of neutrophil apoptosis was inversely proportional to the APACHE II score (The APACHE II score is routinely used to assess disease severity and prognosis in the intensive care unit [24]) (Fig. 3B; R = − 0.4467, p = 0.0371). This showed that patients with ARDS with delayed neutrophil apoptosis are more critical, indicating that the degree of neutrophil apoptosis is related to disease prognosis. Generally, the percentage of neutrophils in peripheral blood can reflect the degree of infection [25]. This shows that when neutrophil apoptosis is delayed, the proportion of neutrophils in the blood increases, and the percentage of neutrophils in peripheral blood can reflect the severity of the infection. Interestingly, cf-DNA/MPO levels in plasma were directly proportional to neutrophils’ percentage (Fig. 3C; R = 0.4104, p = 0.0373). Also we found that the cf-DNA/MPO level was positively correlated with the APACHE II score (Fig. 3D; R = 0.4931, p = 0.0105).

### AT7519, a CDK inhibitor, alleviates LPS-induced ALI in mice

**Fig. 4:**
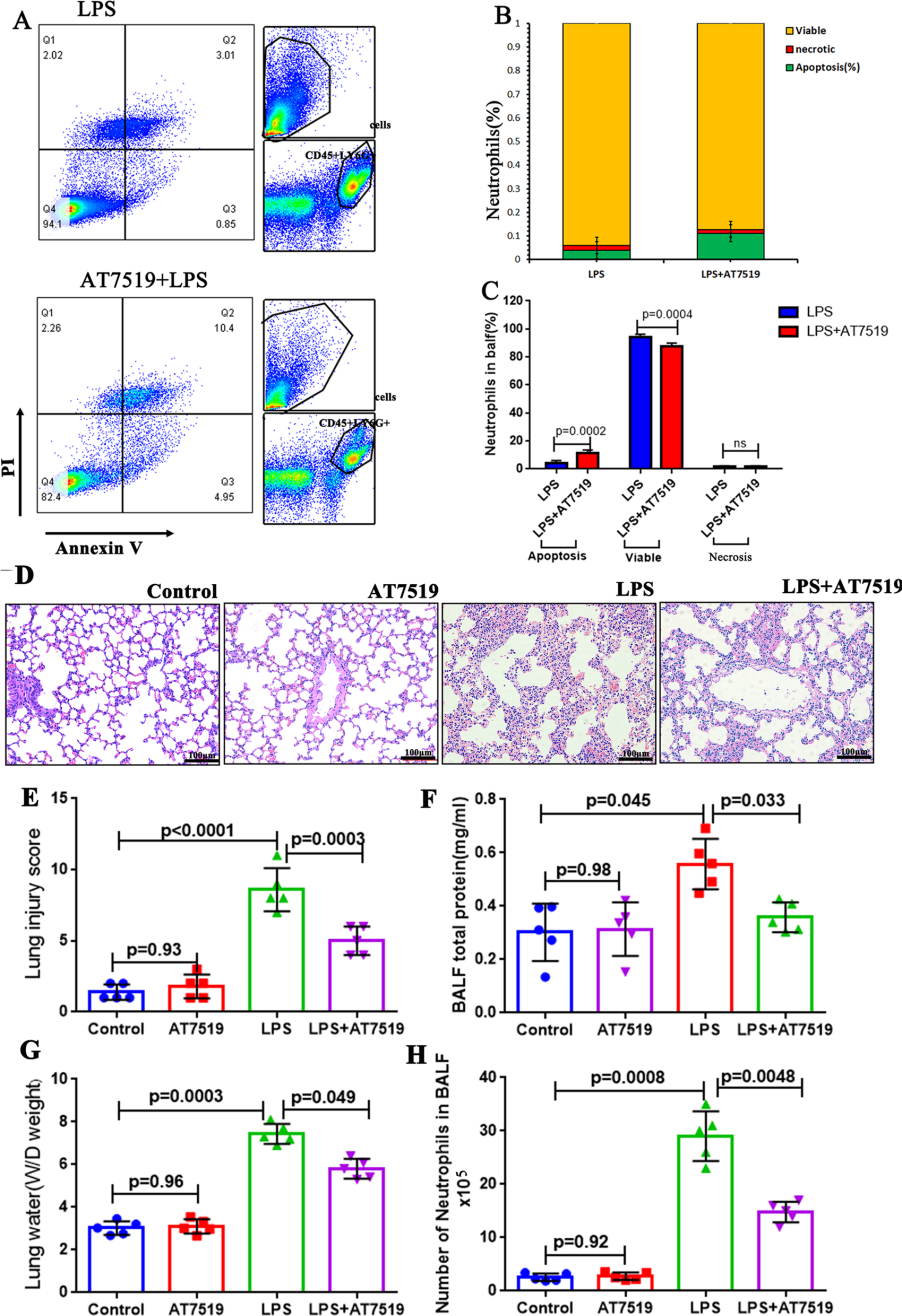
AT7519, a CDK inhibitor, alleviates LPS-induced acute lung injury (ALI) in mice. **A–C** Flow cytometry, n = 5; **D** hematoxylin–eosin (HE) staining, n = 5; **E** Lung injury score, n = 5; **F** bronchoalveolar lavage fluid (BALF) total protein, n = 5; **G** Lung water/dry (W/D) weight, n = 5; **H** Neutrophils in BALF (× 10^5^), n = 5. p values < 0.05 indicated statistical significance

From the above clinical studies, we know that neutrophil apoptosis is delayed in patients with ARDS, and has a negative correlation with NET levels. According to the existing reference, we know that the CDK inhibitor AT7519 can correct neutrophil apoptosis and attenuates inflammation [26], so we conducted animal experiments and divided them into control group, LPS group, and LPS + AT7519 group. First, we collected mouse alveolar lavage fluid, and used the Annexin-V/PI kit to detect the difference in alveolar neutrophil apoptosis (Fig. 4A–C). We found that the neutrophil apoptosis rate in the LPS + AT7519 group was higher than that in the LPS group (p = 0.0002), while the proportion of necrotic cells was not significantly different. There were more viable neutrophils in the LPS group than AT7519 (p = 0.0004). Since AT7519 promotes alveolar neutrophil apoptosis, does it also affect lung tissue injury? Next, we measured the protein concentration in BALF, and retained the lung tissue samples for HE staining. The HE staining showed that pulmonary edema, alveolar congestion, alveolar septal thickening and leukocyte infiltration were more severe in the LPS group than LPS + AT7519 groups (Fig. 4D) and the lung injury score (Fig. 4E; p = 0.0003), total protein concentration in BALF (Fig. 4F; p = 0.033), pulmonary wet/dry (W/D) weights (Fig. 4G; p = 0.049), and number of neutrophils in BALF (Fig. 4H; p = 0.0048) were significantly reduced in the LPS + AT7519 groups compared with those in the LPS group. The above results demonstrate that AT7519 can inhibit lung injury in LPS-induced ALI in mice by promoting neutrophil apoptosis.

### AT7519, a CDK inhibitor, attenuates NETs in LPS-induced ALI

**Fig. 5:**
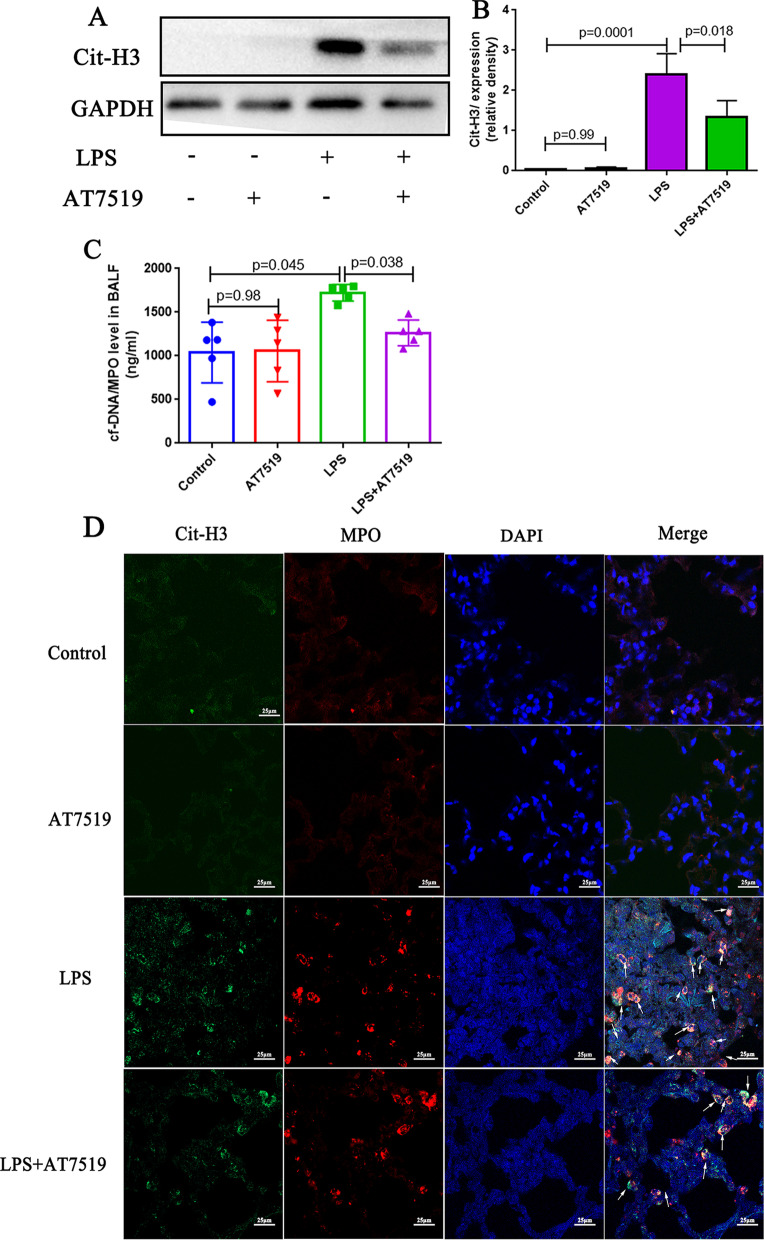
AT7519, a CDK inhibitor, reduces NETs in LPS-induced acute lung injury (ALI). **A**, **B** Western blotting was used to determine the Cit-H3 level, n = 3; The immunoblot data currently presented as separate groups were run on the same gel (with non-essential lanes removed). **C** PicoGreen assay was used to determine the cf-DNA/MPO level in the BALF, n = 5. **D** Confocal microscopy is used to detect MPO/Cit-H3 levels in lung tissue. p values < 0.05 indicated statistical significance

Because clinically data showed that delayed neutrophil apoptosis in patients with ARDS may enhance the production of NETs, And NET levels were related to the severity of the disease. Therefore, we measured the level of NETs between LPS group and LPS + AT7519 group to verify whether the level of NETs will change after promoting neutrophil apoptosis. Figure 5A and B showed that the Cit-H3 level in the LPS group was significantly higher than the control group (p = 0.0001), but in the LPS + AT7519 group, the Cit-H3 level was reduced compared with the LPS group (p = 0.018), and the differences were statistically significant, which was consistent with the confocal result of the MPO/Cit-H3 level in lung tissues (Fig. 5D). And from the results of the PicoGreen assay, we found that the cf-DNA/MPO level in the BALF was higher in the LPS group than in the control group (Fig. 5C; p = 0.045). However, when AT7519 was used to promote neutrophil apoptosis, cf-DNA/MPO levels were correspondingly reduced (Fig. 5C; p = 0.038). The above results indicated that the correction of neutrophil apoptosis is accompanied by a decrease in the levels of NETs in the alveoli, which may be one of the factors that mitigate lung injury.

### AT7519, a CDK inhibitor, reverses neutrophil apoptosis

**Fig. 6:**
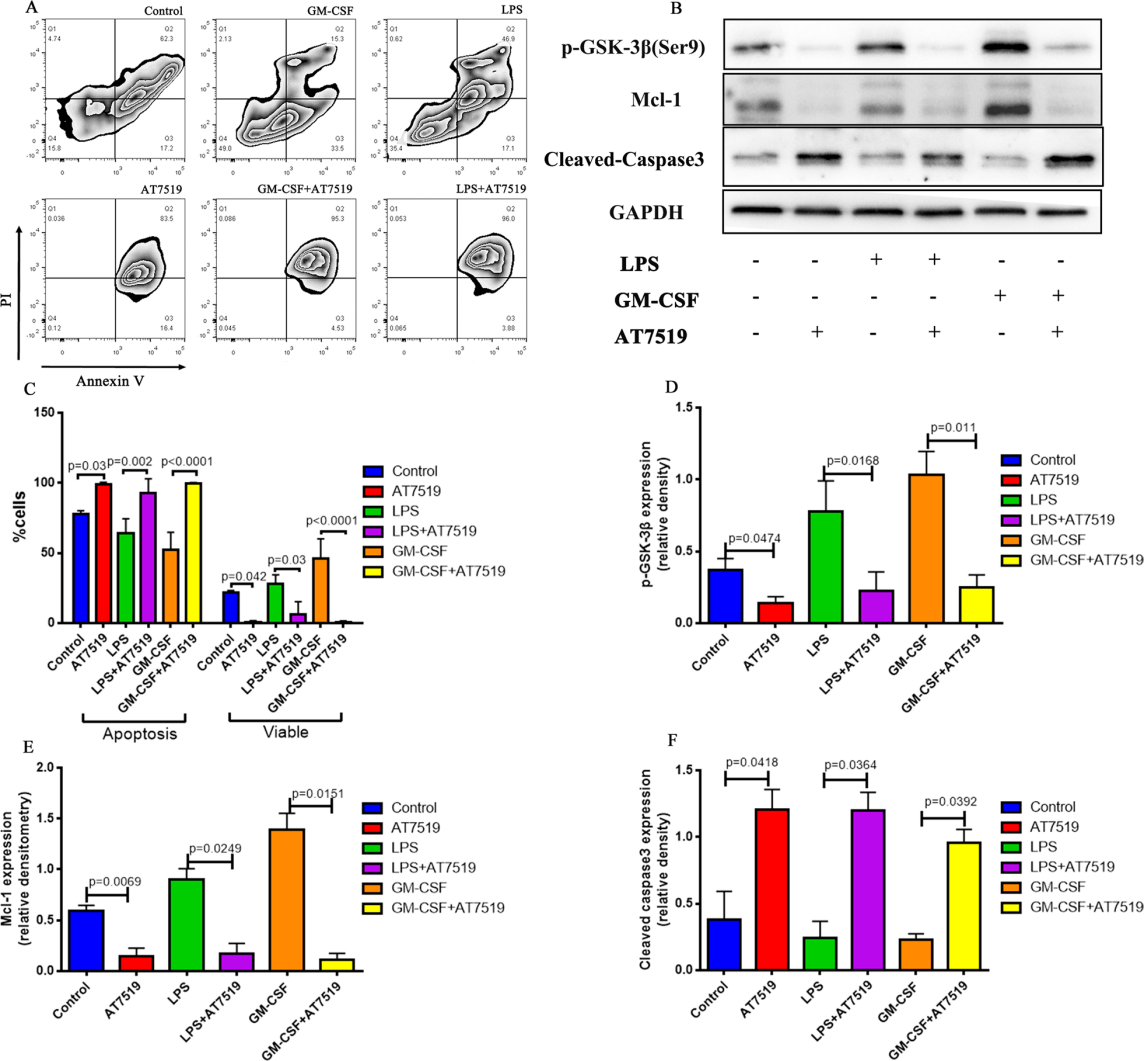
AT7519, a CDK inhibitor, reverses neutrophil apoptosis in vitro. **A**, **B** Flow cytometry. GM-CSF and LPS delayed the neutrophil apoptosis and significantly increased cell viability. After AT7519 was added, the inhibitory effect of GM-CSF and LPS on neutrophil apoptosis was reversed (p < 0.0001, p = 0.002, respectively) and the ability to promote neutrophil survival was also weakened (p < 0.0001, p = 0.03, respectively). n = 3. **C**–**F** Western blot. When adding LPS and GM-CSF into medium, the level of p-GSK-3β (Ser9) increased, but in the presence of AT7519, the level of p-GSK-3β (Ser9) (**D**) was significantly reduced compared with the absence of AT7519, n = 4; changes in Mcl-1 (**E**) expression levels were consistent with that of p-GSK-3β (Ser9), n = 3; Cleaved-caspase 3 (**F**), a marker of apoptosis, increased when the medium contained AT7519, n = 3. The immunoblot data currently presented as separate groups were run on the same gel (with non-essential lanes removed). p values < 0.05 indicated statistical significance

It has been demonstrated in vivo that AT7519 can reverse LPS-induced ALI neutrophil apoptosis. However, there are various cells and various influencing factors in the internal environment of the animals. We cannot know whether AT7519 directly or indirectly affects the lifespan of neutrophils. In order to eliminate the interference of other confounding factors, we further separated the ARDS neutrophils and stimulated them with AT7519 and pro-survival factors, such as GM-CSF (2.5 ng/ml) or LPS (10 ng/ml), for 24 h to explore the possible pro-apoptotic mechanism of AT7519. In flow cytometry (Fig. 6A and B), we observed that GM-CSF and LPS delayed neutrophil apoptosis and significantly increased cell viability. After AT7519 was added, the inhibitory effect of GM-CSF and LPS on neutrophil apoptosis was reversed (p < 0.0001 and p = 0.002, respectively) and the ability to promote neutrophil survival was also weakened (p < 0.0001 and p = 0.03, respectively). Western blotting results (Fig. 6C and D) showed that when AT7519 was added, the level of p-GSK-3β (Ser9) decreased. When LPS and GM-CSF were added to the medium, the level of p-GSK-3β (Ser9) increased, but in the presence of AT7519 the level of p-GSK-3β (Ser9) was significantly reduced compared with the absence of AT7519 (control vs. AT7519 group, p = 0.0474; GM-CSF group vs. GM-CSF + AT7519 group, p = 0.011; LPS vs. LPS + AT7519, p = 0.0168). Simultaneously, we also observed that changes in Mcl-1 expression levels were consistent with those of p-GSK-3β (Ser9) (Fig. 6C and E). Cleaved-caspase 3 (Fig. 6C and F), a marker of apoptosis, exhibited increased expression when the medium contained AT7519 (control vs. AT7519 group, p = 0.0418; GM-CSF group vs. GM-CSF + AT7519 group, p = 0.0364; LPS vs. LPS + AT7519, p = 0.0392). These results indicate that AT7519 can promote neutrophil apoptosis and reverse the anti-apoptotic effects of pro-survival factors such as GM-CSF and LPS, which exist in the ARDS environment.

### Reversed neutrophil apoptosis attenuates NETs in vitro

**Fig. 7:**
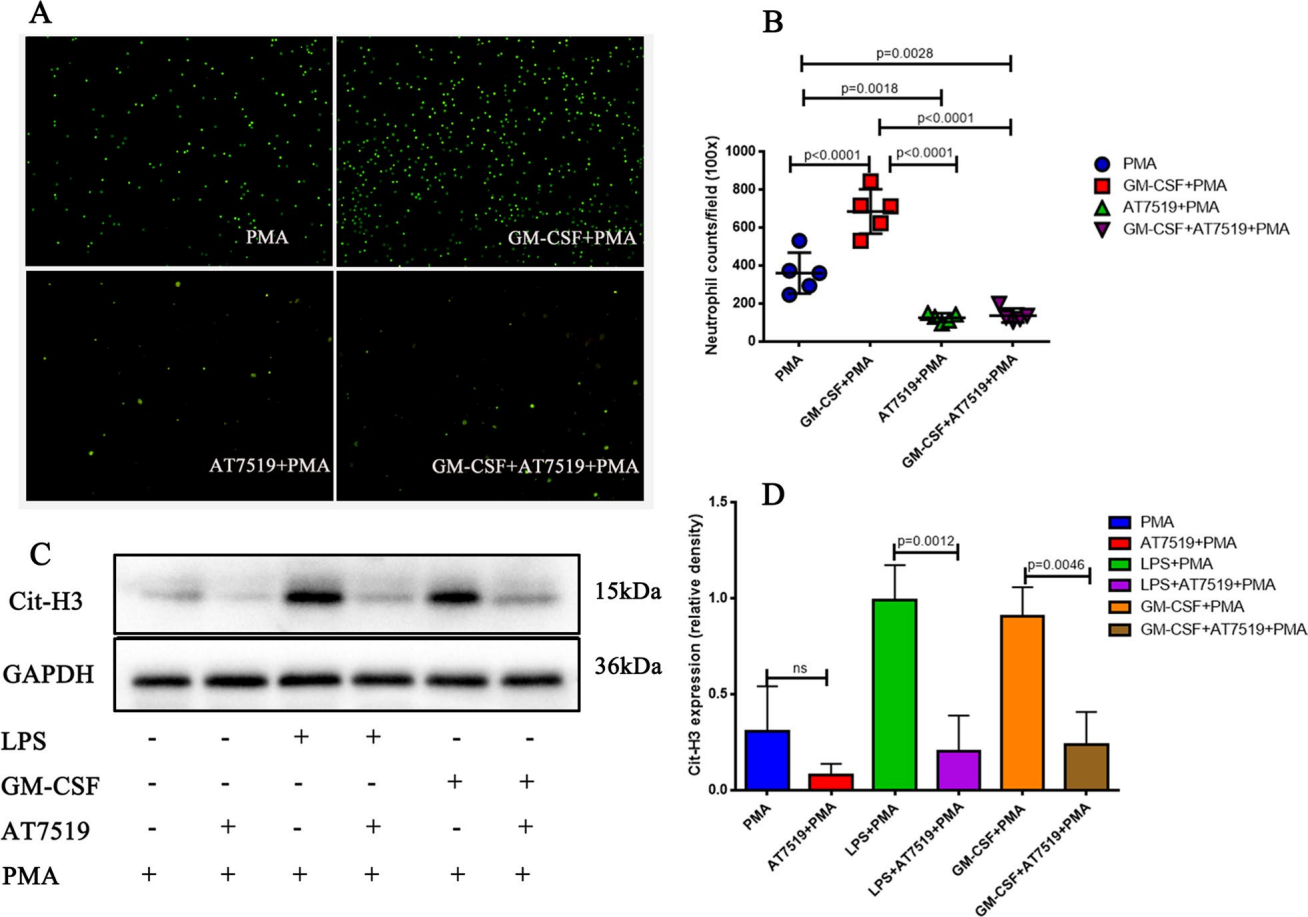
Reversed neutrophil apoptosis reduces neutrophil extracellular traps (NETs) in vitro. **A**, **B** SYTOX Green staining. The stimulation of GM-CSF to ARDS neutrophils further increased NET formation, this effect was reversed by AT7519, n = 5. **C**, **D** Western blot results showed that the Cit-H3 expression was highest in ARDS neutrophils with GM-CSF and LPS, and the situation was reversed by AT7519. The immunoblot data currently presented as separate groups were run on the same gel (with non-essential lanes removed), n = 3. p values < 0.05 indicated statistical significance

Next, we assessed whether adding pro-survival factors to neutrophils would enhance NET formation or induce early apoptosis of neutrophils to reduce NET formation. Neutrophils were cultured for 8 h in the presence of GM-CSF (2.5 ng/ml) or LPS (10 ng/ml) ± AT7519 (1 µM) and then stimulated with PMA (40 nM) for 4 h. We used SYTOX Green staining and western blotting to detect NET levels. The stimulation of GM-CSF to ARDS neutrophils further increased NET formation (Fig. 7A and B, GM-CSF + PMA group vs. PMA group, p < 0.0001); this effect was reversed by AT7519 (Fig. 7A and B; GM-CSF + AT7519 + PMA group vs. GM-CSF + PMA group, p < 0.0001). Moreover, we used western blotting to detect the Cit-H3 protein level and found that Cit-H3 expression was highest in ARDS neutrophils with GM-CSF and LPS, and the situation was reversed by AT7519 stimulation (Fig. 7C and D; GM-CSF + PMA group vs. GM-CSF + AT7519 + PMA group, p = 0.0046, LPS + PMA group vs. LPS + AT7519 + PMA group, p = 0.0012). These results suggested that inducing apoptosis stopped NET formation.

## Discussion

It is recognized that neutrophils are a crucial component of the inflammatory response and play an essential role in the pathogenesis of ARDS [27, 28]. Indeed, the degree and duration of neutrophil activity in the alveolar airspace in ARDS are essential indicators of prognosis [29]. Although the presence of neutrophils in inflammatory tissue does not imply that these cells have a pathogenic effect, we and others have shown that neutrophils in the alveolar airspace are highly sensitized and have a pro-survival phenotype in ARDS, demonstrating enhanced superoxide anion and protease release. The formation of NETs and delayed apoptosis are considered important drivers of lung injury [7, 10, 25].

Previous studies have demonstrated that during the acute phase of ARDS, especially in the first 3 days, GM-CSF levels in the alveoli and blood increased, and during this time, the BALF or serum obtained from patients with ARDS had anti-apoptotic effects on control neutrophils [6, 25]. BALF from patients with ARDS caused by gram-negative bacillus pneumonia contains LPS [30], which is a component of the outer wall of the gram-negative bacterial cell wall, and GM-CSF and LPS are known anti-apoptotic factors [31, 32]. We used GM-CSF and LPS to simulate the internal environment of ARDS by stimulating neutrophils in vitro.

This study found that neutrophils in ARDS caused by gram-negative bacilli pneumonia were experiencing varying degrees of delayed apoptosis, which was consistent with the results of sepsis-related ARDS reported in reference [2]. Moreover, the degree of apoptosis was inversely proportional to the severity of ARDS. In patients with pulmonary cystic fibrosis, there are reports that delayed neutrophil apoptosis allows enhanced NET formation, which can in turn induce inflammation. Furthermore, we observed in our previous studies that NETs could induce the inflammatory phenotype in macrophages, thereby aggravating the inflammatory response and tissue damage [10]. Our study found that reversal of neutrophil apoptosis could reduce NET production, thereby preventing excessive lung tissue damage. cf-DNA/MPO is a component of NETs that indirectly represents the level of NETs, and we found that cf-DNA/MPO levels in the peripheral blood increased when apoptosis was blocked. Combining our previous findings and other reports from the literature, we concluded that the inflammatory response worsened. Therefore, we used the Pearson’s correlation analysis to determine the correlation between the NET level and ARDS severity, and we found that ARDS severity was positively correlated with the NET level. It can be concluded that the level of NETs may be related to the prognosis of patients with ARDS induced by bacterial pneumonia. Maruchi et al. have found that the cf-DNA/MPO levels are related to the severity of organ dysfunction and 28-day mortality in patients with sepsis [33], somewhat similar to our findings. This suggests that maybe we can also detect cf-DNA/MPO levels in the plasma or BALF to diagnose ARDS.

GSK-3β is a critical downstream protein in the PI3K/AKT pathway. Some studies have reported that GSK-3β activity is related to cell activity and that the phosphorylation of GSK-3β serine 9 (inhibits GSK-3β activity) promotes cell survival and prevents apoptosis. Our study found that the anti-apoptotic protein, Mcl-1, was increased in patients with ARDS, although p-GSK-3β (Ser9) was not significant, but we can see that the p-GSK-3β (Ser9) level of some patients was increasing, and increased significantly in in vitro cell experiment level. It can be seen that the p-GSK3β/Mcl-1 pathway is activated in ARDS to inhibit neutrophil apoptosis. A study has shown that the CDK pathway is related to neutrophil apoptosis [14]. Although neutrophils are terminal cells, CDK-4, -6, -7, -9, and other proteins related to cell proliferation and the cycle can be expressed in neutrophils. Also, CDK is homologous to GSK-3β [34] and several CDK inhibitors have been shown to inhibit GSK3 expression [35]. In multiple myeloma, AT7519 induces rapid dephosphorylation of GSK-3β at Ser9, leading to in vitro apoptosis and in vivo antitumor activity, which prolongs survival [18].

CDK inhibitors can improve inflammatory resolution in several lung injury models, including bleomycin-induced, endotoxin-induced, and bacterial-induced lung injuries [14, 36]. Interestingly, in *E. coli*-induced ALI models, administration of CDK inhibitor drugs after the onset of inflammation can increase lung inflammation resolution without adversely reducing the clearance of bacteria. They can also reduce neutrophil apoptosis in patients with sepsis-related ARDS in vitro [2]. Analysis of the clinical data revealed that neutrophil apoptosis delay is related to NET formation. A study has shown that inhibiting the CDK4/6 pathway can inhibit the NET production pathway and cause apoptosis [19]. The inhibition of AKT prevented NETs production and instead switched to caspase-dependent apoptotic pathways [37]. AKT is associated with several proteins related to cell survival, including GSK-3β, P21, and P27 [14]. If the activity of AKT is blocked, the activities of these anti-survival factors will be affected. It has been demonstrated that CDK inhibitors can affect GSK-3β activity [18].

In our research, we demonstrated that AT7519, a CDK inhibitor, promotes neutrophil apoptosis. Concurrently, we also found that the levels of p-GSK-3β (Ser9) and Mcl-1 decreased with the addition of AT7519, accompanied by a significant decrease in a NET generation, irrespective of the presence or absence of pro-survival factors such as GM-CSF or LPS. A CDK pathway protein, an essential survival protein downstream of AKT, or multiple mechanisms may be involved in the relationship between NET generation and neutrophil apoptosis.

As for whether AT7519 affects the recruitment of neutrophils in the LPS-ALI animal model, we have not conducted further studies. However, it has been found that AT7519 does not affect the recruitment of neutrophils (for example, at the injury tissue in the model of zebrafish tailfin injury) [26, 38], but can reduce inflammation by promoting neutrophil apoptosis [36, 38]. Apoptotic neutrophils can be phagocytosis by macrophages. Therefore, we speculate that apoptotic neutrophils being cleared eventually lead to the decrease of neutrophils in the LPS-ALI model.

We acknowledge that this study has several limitations. For example, we mainly investigated the effect of delayed neutrophil apoptosis on NET production in patients with ARDS. However, the specimens we obtained were mainly from the peripheral blood, and only a few samples of neutrophils from some patients were obtained for flow cytometry analysis (Additional file 3: Fig. S1). The number of patient samples obtained in this study was small, and neutrophils were not cultured using human BALF supernatant or human autologous serum. Instead, LPS and GM-CSF were used to mimic the internal environment, which cannot truly represent the body’s inflammatory environment. Also, we did not conduct specific mechanism pathway studies. In future research, we plan to expand the amount of clinical samples, especially the neutrophils in the alveolar lavage fluid. At the same time, we will perform RNA-seq on the collected neutrophil samples to screen out the differential pathways, and verify them in vitro.

## Conclusions

In summary, neutrophil apoptosis has been found to be inversely proportional to the severity of ARDS, the CDK inhibitor AT7519 can reverse this phenomenon in animal experiments and in vitro, which is consistent with the findings in the references for sepsis-associated ARDS [2, 30], however, the difference is that our study mainly focuses on ARDS caused by gram-negative bacilli pneumonia. Besides, we also novelly revealed that delayed neutrophil apoptosis enhances NET formation in patients with ARDS, and promoting apoptosis can reduce NETs formation, thereby effectively controlling the inflammatory response in lung tissue. Because our previous research has demonstrated that excessive NETs can promote macrophage polarization to M1 phenotype and aggravate the inflammatory response [10], there may be a relationship between neutrophil apoptosis, NET formation, and macrophage polarization. Neutrophil apoptosis may serve as a critical target in the regulation of other inflammatory responses. Neutrophil apoptosis induced by CDK inhibitors may reverse NET formation, maintain the M1/M2 polarization balance, and can be adopted as an essential approach for the targeted treatment of acute inflammation in ARDS.

## Supplementary Information

**Additional file 1: Table S1.** Characteristics of ARDS patients and healthy controls whose neutrophil apoptosis has been detected.

**Additional file 2: Table S2.** Details of the 22 ARDS patients.

**Additional file 3: Figure S1. **Apoptosis of neutrophils in BALF of 24 h.

## Data Availability

The datasets generated and/or analysed during the current study are not publicly available due to patient privacy but are available from the corresponding author on reasonable request.

## Abbreviations

ARDS: Acute respiratory distress syndrome
NETs: Neutrophil extracellular traps
LPS: Lipopolysaccharide-induced
ALI: Acute lung injury
BALF: Bronchoalveolar lavage fluid
GM-CSF: Granulocyte-macrophage colony-stimulating factor
TNF: Tumor necrosis factor
Mcl-1: Myeloid cell leukemia-1
RICU: Respiratory intensive care unit
DPBS: Dulbecco’s phosphate-buffered saline
FBS: Fetal bovine serum
PMA: Para-methoxyamphetamine
cf-DNA: Cell-free DNA
MPO: Myeloperoxidase
EDTA: Ethylenediamine tetraacetic acid
HE: Hematoxylin–eosin
SE: Standard error
