# Supplementary material for: Delayed neutrophil apoptosis may enhance NET formation in ARDS
Source: Respir Res. 2022 Jun 13;23:155. doi: 10.1186/s12931-022-02065-y (PMC9190136; doi:10.1186/s12931-022-02065-y)
Supplement: Supplementary file 1 — Additional file 1: Table S1. Characteristics of ARDS patients and healthy controls whose neutrophil apoptosis has been detected. [file 12931_2022_2065_MOESM1_ESM.docx]

| Parameter | ARDS(n=22) | Control(n=13) | P value |
| --- | --- | --- | --- |
| Age, years | 65.46 ± 16.02 | 58.15±16.50 | 0.63 |
| Gender, male/female | 22, 13/9 | 13,6/7 | 0.713 |
| PaO2 /FiO2 ratio* | - | - | - |
| 200–300 mmHg (mild) | 11 | - | - |
| APACHE II | 11.00 ± 4.775 | - | - |
| 100–200 mmHg (moderate) | 9 | - | - |
| APACHE II | 15 ± 6.245 | - | - |
| ≤100 mmHg (severe) | 2 | - | - |
| APACHE II | 20 ± 5.657 | - | - |
| Cause |  | - | - |
| Direct (Severe pneumonia) | 22 | - | - |
| Indirect | 0 | - | - |
| pathogen |  | - | - |
| Acinetobacter baumannii | 6 | - | - |
| E.coli | 3 | - | - |
| Klebsiella pneumoniae | 3 | - | - |
| Pseudomonas aeruginosa | 5 | - | - |
| Acinetobacter pittii | 1 | - | - |
| Haemophilus influenzae | 2 | - | - |
| Burkholderia cepacia | 1 | - | - |
| Stenotrophomonas maltophilia | 1 | - | - |
| Antibiotics | - | - | - |
| Quinolones | 8 | - | - |
| Carbapenems | 9 | - | - |
| Cephalosporins | 1 | - | - |
| Penicillins | 7 | - | - |
| Combination medication  (at least two antibiotics) | 4 | - | - |

**Table S1 Characteristics of ARDS patients and healthy controls whose neutrophil apoptosis has been detected**

**Table S1.** Data are presented as mean ± SD or n (%), *Identical PaO2/FiO2 cutoff values are used in the Berlin Definition of ARDS severity[1].
