# Supplementary material for: Delayed neutrophil apoptosis may enhance NET formation in ARDS
Source: Respir Res. 2022 Jun 13;23:155. doi: 10.1186/s12931-022-02065-y (PMC9190136; doi:10.1186/s12931-022-02065-y)
Supplement: Supplementary file 2 — Additional file 2: Table S2. Details of the 22 ARDS patients. [file 12931_2022_2065_MOESM2_ESM.docx]

| **Patient number** | **Age**  **(years old)** | **cf-DNA/MPO**  **(ng/ml)** | **Apoptosis rate(%)** | **PaO2 /FiO 2 ratio(mmHg)** | **Apache II score** |
| --- | --- | --- | --- | --- | --- |
| 1 | 76 | 160.6933 | 76.9 | 110 | 8 |
| 2 | 53 | 211.2551 | 77.26 | 284.8484848 | 7 |
| 3 | 76 | 146.7607 | 75.46 | 219.6721311 | 8 |
| 4 | 64 | 233.2227 | 73.8 | 222.8571429 | 8 |
| 5 | 58 | 295.6371 | 72.48 | 112.5 | 12 |
| 6 | 68 | 239.6815 | 71.36 | 238 | 20 |
| 7 | 53 | 199.8151 | 70.73 | 117.1428571 | 26 |
| 8 | 80 | 155.9742 | 70.35 | 255 | 7 |
| 9 | 77 | 327.0978 | 69.21 | 273.4693878 | 12 |
| 10 | 75 | 167.9966 | 64.75 | 281.25 | 14 |
| 11 | 81 | 153.9517 | 64.7 | 219.1489362 | 4 |
| 12 | 23 | 172.3787 | 61.37 | 289 | 15 |
| 13 | 67 | 220.8056 | 57.4 | 120 | 10 |
| 14 | 72 | 149.9067 | 55.9 | 289.0909091 | 18 |
| 15 | 79 | 350.8056 | 51.67 | 121.6666667 | 16 |
| 16 | 55 | 373.1652 | 51.3 | 153.9516854 | 31 |
| 17 | 82 | 252.8281 | 42.53 | 160 | 22 |
| 18 | 60 | 489.682 | 38.77 | 95 | 21 |
| 19 | 21 | 384.2249 | 32.22 | 160.9756098 | 18 |
| 20 | 52 | 217.8843 | 22.7 | 89 | 14 |
| 21 | 70 | 304.8506 | 16.94 | 183.7837838 | 16 |
| 22 | 80 | 201.5969 | 88.9 | 296 | 7 |

**Table S2 Details of the 22 ARDS patients**
