# Supplementary figures and images for: Delayed neutrophil apoptosis may enhance NET formation in ARDS
Source: Respir Res. 2022 Jun 13;23:155. doi: 10.1186/s12931-022-02065-y (PMC9190136; doi:10.1186/s12931-022-02065-y)

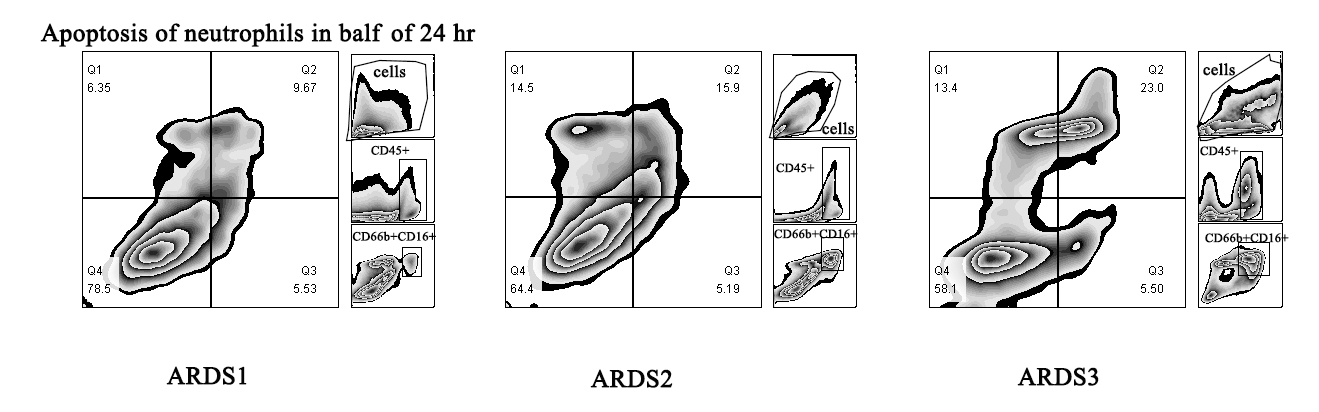

Supplement: Supplementary file 3 — Additional file 3: Figure S1. Apoptosis of neutrophils in BALF of 24 h. [file 12931_2022_2065_MOESM3_ESM.tif]
